# Monitoring microcirculatory blood flow with a new sublingual tonometer in a porcine model of haemorrhagic shock

**DOI:** 10.1186/cc12148

**Published:** 2013-03-19

**Authors:** P Palagyi, J Kaszaki, Z Molnar

**Affiliations:** 1 University of Szeged, Hungary

## Introduction

Tissue capnometry has been used to assess organ perfusion but it is not generally available at the bedside [1,2]. Our aim was to test a new sublingual capillary tonometer in haemorrhagic shock.

## Methods

Thirty-six mini-pigs were anaesthetised, ventilated and divided into sham operated and shock groups. Instrumentation included: intestinal and sublingual tonometry, haemodynamic monitoring (PiCCO; Pulsion, Germany) and orthogonal polarization spectroscopy (OPS). After baseline measurements (T0) haemorrhagic shock was induced and maintained by reducing mean arterial pressure (MAP) to ~40 mmHg for 60 minutes. Measurements were repeated every 30 minutes (T1 to T6). Fluid resuscitation started after T2 aiming to increase MAP to 75% of the baseline value. OPS imaging was performed at T0, T2 and T6. Data are presented as the median (interquartile range), for statistical analysis Friedmann ANOVA, Mann-Whitney and Spearman tests were used as appropriate.

## Results

Bleeding resulted in a significant decrease in MAP and cardiac index, and an increase in heart rate. Macrohaemodynamic changes were accompanied by significant changes in red blood cell velocity (RBCV) and a significant increase in the intestinal and sublingual mucosal-to-arterial carbon dioxide partial pressure difference (PCO_2 _gap): from 4 (2 to 11) to 30 (23 to 37) mmHg in the sublingual, and from 25 (17 to 31) to 50 (33 to 64) mmHg in the ileum. RBCV decreased from 1,075 (945 to 1,139) to 520 (449 to 621) μm/second in the sublingual area, and from 646 (596 to 712) to 419 (350 to 451) μm/second in the ileum. There was significant correlation between RBCV and PCO_2 _gap in sublingual and intestinal regions alike: *r *= -0.58; *r *= -0.71, *P <*0.0001, respectively.

## Conclusion

In this model of haemorrhagic shock, sublingual PCO_2 _gap showed good correlation with RBCV, suggesting that this new sublingual capillary tonometer may be an appropriate tool for monitoring microcirculation at the bedside.
